# Exploring IRES Region Accessibility by Interference of Foot-and-Mouth Disease Virus Infectivity

**DOI:** 10.1371/journal.pone.0041382

**Published:** 2012-07-18

**Authors:** Teodoro Fajardo, Maria Flora Rosas, Francisco Sobrino, Encarnacion Martinez-Salas

**Affiliations:** Centro de Biología Molecular Severo Ochoa, Consejo Superior de Investigaciones Cientificas-Universidad Autonoma de Madrid, Madrid, Spain; Pohang University of Science and Technology, Republic of Korea

## Abstract

Translation initiation of picornavirus RNA is driven by an internal ribosome entry site (IRES) element located upstream of the initiator codon. RNA structure organization as well as RNA-protein interaction plays a fundamental role in internal initiation. IRES activity has been mainly analyzed in the context of reporter genes, lacking regions of the viral genome potentially affecting translation efficiency. With the aim to understand the vulnerability of the IRES and translation start region to small molecules in the context of the viral genome, we designed a set of customized RNase-resistant 2′O-methyl antisense oligoribonucleotides (2′OMe AONs) based on RNA structure data. These AONs were then used to monitor their capacity to interfere viral RNA translation, and thus, to inhibit virus yield. Foot-and-mouth disease virus (FMDV) RNA translation can be initiated at two in-frame AUG codons. We show here that a 2′OMe AON complementary to AUG2 inhibited viral multiplication more efficiently than the one that targeted AUG1. Furthermore, the response of the viral RNA to AONs targeting the IRES region denoted important differences between tissue culture cells and cell-free systems, reinforcing the need to analyze viral RNA response in living cells. Importantly, we have identified four specific motifs within the IRES element that are targets for viral inhibitors both in tissue culture cells and in cell-free systems. The identified targets define accessible regions to small molecules, which disturb either the RNA structural organization or the RNA-protein interactions needed to initiate translation in FMDV RNA.

## Introduction

**Figure 1:**
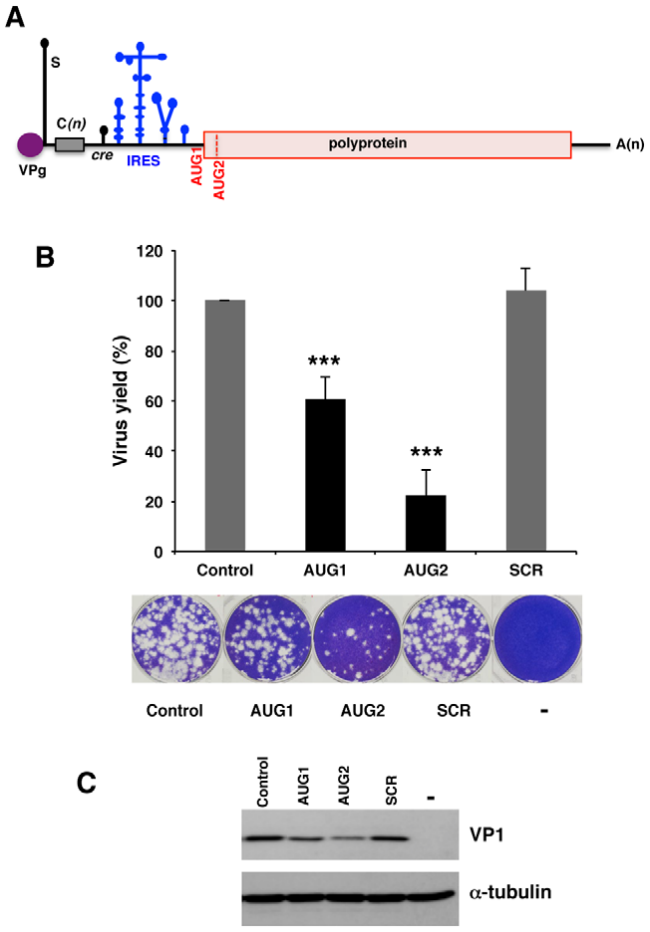
Differential effect of 2′OMe AONs targeting the initiator codons on virus yield. (A). Schematic representation the foot-and-mouth viral genome; the position of the IRES and the initiator codons, AUG1 and AUG2 is depicted. (B). Changes in viral yield induced by AONs targeting AUG1 and AUG2 initiation codons. *In vitro* synthetized FMDV RNA (50 pg) was annealed with the indicated concentrations of AONs AUG1, AUG2 or the scramble SCR, prior to transfect confluent BHK-21 cell monolayers in duplicate. Virus yield was determined using fresh cells monolayers as the number of plaque forming units (PFU)/ml in the supernatant 24 hpt as described in Material and Methods, made relative to the control RNA which was set at 100%. Values represent the mean and standard deviation of triplicate assays (*** *P*<0.01). Representative examples of viral plaques stained with crystal violet are shown. (C). Western blot of viral protein VP1 accumulated in transfected BHK-21cells 24 hpt; anti-tubulin was used as loading control.

Foot-and-mouth disease virus (FMDV) is the prototype of the aphthovirus genus of the *Picornaviridae* family. FMDV causes a contagious disease that affects cattle and a large number of wild cloven-hoofed ungulates [1], [2]. The viral genome consists of a positive sense, single-stranded RNA of about 8500 nts, encoding a single polyprotein, flanked by a heavily structured untranslated region (UTR) at the 5′end (Fig. 1A). Upon entry into the cell, the viral open reading frame is translated into a polyprotein that is proteolytically processed by virus-encoded proteases [3]. In common with all picornaviruses, the FMDV genome does not contain the cap structure (m^7^GpppN) typically present in cellular mRNAs at its 5′ end. Instead, a viral protein (VPg) is covalently linked to the 5′end of the viral RNA [4]. The 5′UTR comprises a long hairpin (termed S), a poly(C) tract of variable length, a variable region folding as two to four pseudoknots, the *cis*-replication element (*cre*), and the internal ribosome entry site (IRES) element that mediates cap-independent translation of the viral RNA [5], [6], [7]. The 3′UTR consists of a region of about 90 nt and a poly(A) tail, which stimulate IRES activity and participates in viral RNA replication [8], [9]. Replication of the viral genome occurs in the cytoplasm of infected cells. Polyprotein synthesis can start at two in-frame AUG codons separated by 84 nt [10]. Both AUGs are used as start codons for viral protein synthesis, although the second one is preferentially utilized in a variety of assays [11], [12].

Picornavirus IRESes are *cis*-acting elements that recruit the 40S ribosomal subunits to the mRNA with the help of cellular *trans*-acting factors [13]. The FMDV IRES is distributed in structural domains (Fig. 1A) with the first 21 nt (domain 1) forming part of the *cre* element [14]. Domain 2 includes a polypyrimidine tract (UCUUU) that provides a polypyrimidine tract-binding protein (PTB) binding site [15]. Domain 3 consists of 210 nts, the most apical region of which is arranged as a cruciform structure that plays a crucial role in RNA-RNA interaction [16], [17]. The proximal part is organized as a base-paired structure interrupted with bulges that include several non-canonical base pairs and a helical structure essential for IRES activity [18], [19]. Domain 4, which is organized into two hairpin-loops with A-rich internal bulges conserved between FMDV and encephalomyocarditis virus (EMCV) [20] is responsible for the interaction with the translation initiation factor eIF4G [21], [22], an essential step in FMDV and EMCV IRES-dependent translation initiation. Domain 5 is composed of a conserved hairpin-loop and a polypyrimidine-rich tract preceding the first functional AUG codon. Specific RNA motifs located in domains 2, 4 and 5 are responsible for the interaction with cellular proteins such as PTB, eIF4G, eIF3 and eIF4B, amongst other host factors controlling internal initiation [23].

The FMDV IRES showed significant differences in the accessibility *in vitro* and *in vivo* to dimethyl sulfate (DMS) [24], a reagent that is permeable to cell membranes and reacts with unpaired bases in the RNA structure. The differences in the accessibility of the IRES to DMS suggested that the IRES region adopts a different conformation in the cell cytoplasm compared to naked RNA. Similarly, results of UV-crosslinked amino-methyl psoralen-treated cells showed a local reorganization of RNA structure. Recent Selective 2′Hydroxyl Acylation analyzed by Primer Extension (SHAPE) structural analysis revealed a modular organization of the IRES region [25]. Additionally, clusters of SHAPE reactive nts indicated the presence of flexible regions, hairpin-loops and internal bulges within the IRES domains [26]. Accessibility to the FMDV IRES was also studied through hybridization of fluorescent-labeled IRES transcripts with complementary oligodeoxynucleotides printed on microarrays. It is worth noting that the accessible regions in the microarray were also reactive to SHAPE probing, except for specific nts within the apical region of domain 3, around the GNRA (where N is any nucleotide and R is a purine) stem-loop [26].

Antisense oligonucleotides are single-stranded sequences that form a hybrid with their complementary RNA via Watson-Crick base pairing. The resulting hybrid can block gene expression by various mechanisms, depending on the chemical composition of the oligonucleotide and location of the hybrid. The strength of the hybrid depends on factors such as thermodynamic stability, the secondary structure of the target mRNA, and the proximity of the hybridization site to functional motifs on the designated transcript such as translational start site. Unmodified oligonucleotides are unstable within cells due to rapid nuclease degradation. By contrast, phosphorothioate [27] and 2′O-methyl oligoribonucleotides, carrying the 2′-OH residue of the ribose molecule replaced by a methyl group, are resistant to degradation by cellular nucleases [28]. 2′O-methyl antisense oligoribonucleotides (2′OMe AONs) form high melting temperature heteroduplexes with targeted mRNA [29] and induce antisense effect by a non-RNase H-dependent mechanism [30], [31]. Earlier studies have been conducted to investigate the use of antisense transcripts to inhibit FMDV viral gene expression directed against the functional AUGs [32] and to both, the 5′ and 3′UTR [33], [34]. An independent study using antisense morpholino oligomers targeting the viral RNA start codons also showed the capacity of these small molecules to inhibit viral FMDV multiplication [35]. The latter study showed controversial results with a previous work [36], in which phosphorothioate antisense oligodeoxynucleotides complementary to AUG2 inhibited virus multiplication with greater effect than those complementary to AUG1. Despite all these studies, a deep analysis of molecules targeting the entire IRES element to inhibit viral RNA expression in cells and *in vitro* was lacking.

Here, by taking advantage of the RNA structural analysis of the IRES-AUG region [12], [16]–[18], [23]–[26], we have designed a set of customized 2′OMe AONs to monitor the accessibility of FMDV in the context of full length RNA transcribed from an infectious cDNA clone. The results indicate that the AON targeting AUG2 inhibited FMDV viral multiplication more efficiently than that which targeted AUG1 in susceptible cells. By contrast, the results obtained using cell-free systems indicated that AUG1 was the best target to inhibit translation. Remarkably, four AONs (183, 207, 432 and 452) complementary to conserved motifs within the apical region of domain 3 and domain 5, that play key roles in internal initiation [17], [37], [38], were potent inhibitors in both systems, revealing accessible IRES regions which are candidate targets for small molecules interfering viral infectivity. Furthermore, the accessibility of different regions of the IRES to 2′OMe AONs exhibited important differences when comparing the results obtained in RNA-transfected cells or *in vitro* translation of the same RNA. These differences, which are likely due to the composition of the cell cytoplasm and the cell-free system, emphasize the need to use living cells to measure the inhibitory capacity of small molecules.

## Results

### Differential inhibition of FMDV RNA infectivity by 2′O-methyl-antisense oligonucleotides targeting AUG1 and AUG2 in tissue culture cells

FMDV RNA translation initiation is peculiar in that protein synthesis can be initiated at two start codons, AUG1 and AUG2 (Fig. 1A). While both AUGs are fully conserved in field isolates, AUG2 is used more efficiently than AUG1 in infected cells [11], [39]. Earlier works have addressed the study of AUG codon usage driven by the FMDV IRES using reporter genes [11], [12], [40], [41]. However, little information was available concerning the influence of the complete viral RNA sequence on the accessibility of the translation start region to small molecules.

To fill this gap, we made use of an infectious cDNA clone [42] to study the capacity of 2′OMe AONs to inhibit FMDV RNA translation in BHK-21 cells. To ascertain the optimum inhibitory conditions, a concentration range (1–20 nM) of three oligonucleotides, AUG1, AUG2 and SCR (harboring a scrambled sequence, Table 1) were annealed to FMDV RNA for 20 min at 37°C prior to transfection of BHK-21 cells. Virus yield was determined 24 hpt relative to a parallel assay conducted without AONs (Fig. S1). Inhibition of FMDV titer was dose-dependent and sequence-specific, being 10 or 20 nM equally efficient in inhibiting the viral titer. Thus, 10 nM was used in all subsequent assays. Furthermore, no effect on the transfection efficiency of the FMDV RNA was observed in the presence of increasing amounts of SCR AON relative to the FMDV RNA alone (Fig. S1); in addition no toxicity was observed when cells were incubated with any of these AONs during 48 hr by cell staining.

**Table 1:** 2′O-methyl Antisense Oligoribonucleotides.

Name	Length	Target region	Sequence (5′–3′)	GC	Tm
40	15	D2: 40–26	mUmUmCmAAGUUGCAmCmGmUmU	40	47
55	16	D2: 55–40	mAmAmGmACCAGGCGGmAmGmUmU	56	57
66	16	D2: 66–51	mCmUmAmGACCUGGAAmAmGmAmC	50	46
83	15	D2: 83–68	mUmAmCmAAAGUGUUmAmCmCmC	40	47
104	19	D3: 104–85	mCmGmAmGCGUGGAGCCAAAmCmAmCmA	60	60
118	14	D3: 118–105	mAmCmUmCGCCAGUmGmGmAmU	57	59
135	18	D3: 135–118	mAmCmAmGUGCUGUUACUmAmAmCmA	39	55
154	18	D3: 154–138	mUmCmAmUGCUCCGCUACmGmAmAmG	56	58
164	14	D3: 164–150	mCmCmAmCGGCCGUmCmAmUmG	71	54
176	14	D3: 176–163	mAmAmGmGAGGAGUmUmCmCmC	57	56
183	17	D3: 183–16717nt	mUmGmUmUACCAAGGAGmGmAmGmU	47	46
192	15	D3: 192–178	mGmUmGmGGUCCUUGmUmUmAmC	54	46
207	17	D3: 207–191	mGmUmGmGCUUUUGGCCmCmCmGmU	65	64
213	15	D3: 213–199	mGmUmGmGGCGUGGCmUmUmUmU	60	53
226	17	D3: 226–210	mAmUmGACGGGCCCGUmGmUmGmG	71	58
247	16	D3: 247–233	mUmCmGmCCGUGCUGGmGmGmUmU	69	61
265	17	D3: 265–249	mUmGmGmGUUUCGCAGUmAmAmAmG	47	56
282	18	D3: 282–265	mUmCmAmAUGUCACUUUAmAmAmGmU	27	45
300	17	D3: 300–284	mAmGmUmGUGUGGGUACmCmAmGmU	53	62
317	17	D4: 317–301	mAmUmCmCUUAGCCUGUmCmAmCmC	53	62
331	16	D4: 331–317	mGmGmGmUACCUGAAGmGmGmCmA	63	60
349	18	D4: 349–334	mAmGmUmGUCGCGUGUUAmCmCmUmC	56	61
360	18	D4: 361–344	mUmCmAmGAUCCCGAGUGmUmCmGmC	61	63
384	18	D4: 384–367	mUmUmAmUAGAAGCCCCAmGmUmCmC	50	56
397	18	D4: 397–378	mAmAmCmCGAGCGCUUUUmAmUmAmG	44	53
407	16	D4: 407–392	mGmAmAmGCUUUUUAAmAmCmCmG	38	44
419	17	D4: 419–404	mUmAmUmUCAGGCAUAGmAmAmGmC	41	49
432	17	D5: 432–415	mCmCmUmCCGGUCACCUmAmUmUmC	59	59
452	19	D5: 452–433	mUmUmGmUAAAGGAAAGGUmGmCmCmG	47	48
AUG AUG	18	luciferase AUG	mCmUmUmCCAUUUUACCAmAmCmAmG	39	49
AUG1 AUG1	19	FMDV AUG1	mCmAmGmUUGUAUUCAUAGmGmGmUmC	42	52
AUG2	18	FMDV AUG2	mGmAmAmUUCCAUUUUUCmCmUmGmC	39	52
SCR	16	Random sequence	mUmGmCmAGCUGACAGmUmGmUmA	50	57

2-O′methyl modifications are designated with a letter ¨m̈. GC, content of GC, Tm, melting temperature.

FMDV multiplication was inhibited in the presence of both, AUG1 and AUG2 AONs (Fig. 1B). Differences in FMDV yield inhibition induced by AONs were analyzed by a paired two-sided Student t-test; differences were considered significant when *P*<0.05. Overall, in agreement with the *P* values obtained in the t-test, 60% was the threshold separating inhibitor from non-inhibitor molecules. The oligonucleotide SCR had no effect on FMDV RNA infectivity (Fig. 1B), demonstrating that the impact of AUG1 and AUG2 AONs on viral gene expression was sequence specific. Moreover, the decrease of virus yield induced by AUG2 (22% relative to the control assay) was 3-fold higher than that of AUG1 (60% of the control RNA), indicating that the best target for inhibition of viral replication is by blocking the AUG2 region.

To confirm the inhibition of viral protein synthesis by AUG1 and AUG2 AONs, a western blot against the VP1 structural protein present in cytoplasmic extracts of BHK-21 transfected cells prepared prior to cell detachment was performed (Fig. 1C). In comparison to cells transfected with FMDV RNA incubated with the SCR AON, or the control without any AON, the intensity of VP1 confirmed that there was a strong inhibition of intracellular viral protein synthesized and accumulated 24 hpt in the presence of AUG2.

### Response of FMDV RNA to 2′OMe AONs targeting the initiator codons using in vitro translation systems

**Figure 2:**
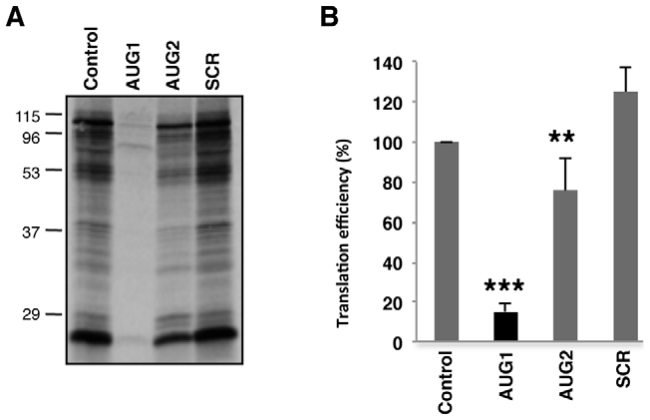
Effect of 2′OMe AONs targeting the initiator AUGs of FMDV RNA in in vitro translation efficiency. (A). Autoradiograph of translation products obtained from *in vitro* synthetized FMDV RNA annealed, or not, with the indicated AONs and incubated with reticulocyte lysates. Translation assays were conducted as described in Material and Methods. The position of molecular weight markers is indicated on the left. (B). The intensity of the lanes were determined by densitometry and made relative to the total intensity of the lane without any AON, which was set at 100%. Data corresponds to the mean and standard deviation of the mean of three independent assays (*** *P*<0.01; ** *P*<0.05). A black bar denotes inhibitory capacity relative to the control translation reaction.

Cell-free systems are often used to determine translation efficiency. Thus, to measure the ability of 2′OMe AONs to inhibit protein synthesis in the context of the complete viral RNA, FMDV RNA preincubated with SCR, AUG1 or AUG2 oligonucleotides under the same annealing conditions used in the RNA transfection assays was used to program rabbit reticulocyte lysates (RRL) translation. The control RNA, without any AON, was efficiently translated, as expected. Analysis of the translation efficiency of FMDV RNA annealed to AONs AUG1 or AUG2 revealed a marked difference in protein synthesis (Fig. 2A). In contrast to the data observed in transfected cells, translation efficiency in the presence of AUG1 (15%) was significantly lower than that observed in the presence of AUG2 (Fig. 2B). Translation of viral proteins in the presence of AUG2 (75% of the value observed with the control RNA alone) was non-inhibitory (*P*<0.01). No inhibition was observed in the presence of SCR, again showing a sequence-specific effect of AONs. Thus, we conclude that there is a contrast between cell-free systems and transfected cells in the response to AON interference depending upon the targeted initiation codon.

### Impact of 2′OMe AONs targeting the IRES region on the FMDV RNA infectivity

**Figure 3:**
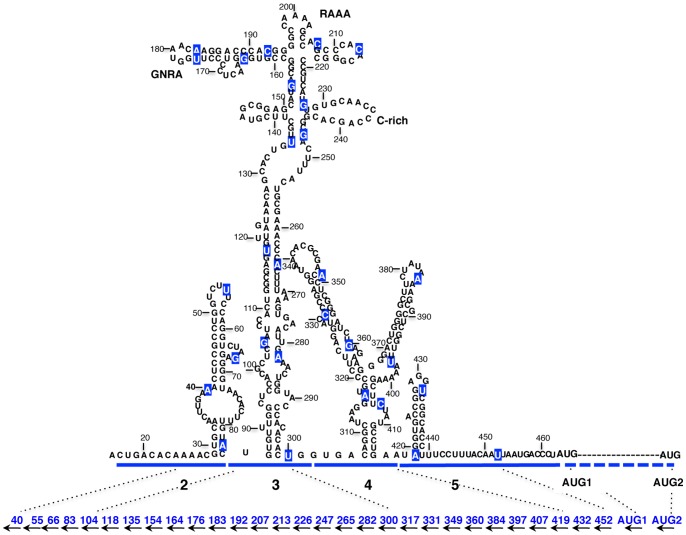
Secondary RNA structure of the FMDV IRES region. The modular organization of the IRES element in domains (1–2, 3, 4, and 5) is indicated. The first 20 nt of the IRES belong to the right arm of *cre* (Fig. 1A); nucleotide numbering has been maintained for consistency with previous works. The position of conserved RNA motifs refer to in the text are indicated. In-frame initiator codons AUG1 and AUG2 are separated by 84 nt (dashed line). Blue-boxed nucleotides depict the annealing nucleotide position of the 3′end of each antisense oligonucleotide. The approximate annealing position of the customized 2′O-Me AONs in the linear IRES sequence is depicted in the bottom. AON sequences are described in Table 1.

**Figure 4:**
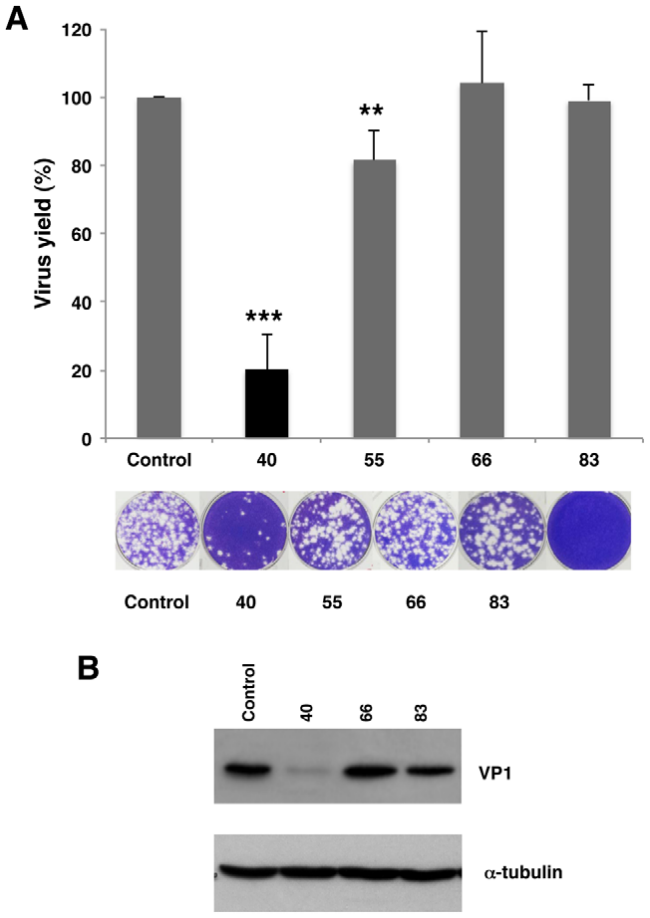
Analysis of the effect of 2′OMe AONs targeting domain 2 of the IRES in virus yield. (A) *In vitro* synthetized FMDV RNA was annealed with oligonucleotides 40, 55, 66 and 83 (see Fig. 3 and Table 1) and the scramble (SCR), prior to transfect confluent BHK-21 cell monolayers in duplicate. Virus yield was determined as PFU/ml in the supernatant 24 hpt, and made relative to the value obtained in the control RNA without AONs. Values represent the mean and standard deviation of triplicate assays (*** *P*<0.01; ** *P*<0.05). Representative examples of viral plaques stained with crystal violet are shown. (B). Western blot of viral protein VP1 accumulated in BHK-21cells 24 hpt; anti-tubulin was used as a loading control.

The differential response of the functional start codons in the two systems used to analyze FMDV RNA translation prompted us to assess the impact of a panel of customized 2′OMe AONs (Table 1) targeted to the entire IRES region (Fig. 3). AONs design was carried out taking into account the structural analysis of the IRES region [16]–[18], [25]–[26]. The capacity of each oligonucleotide to inhibit viral yield, measured by plaque forming units (PFU), was determined 24 hpt. A significant inhibition was noted in the 5′region of the IRES, where virus yield fell to 20% in the presence of the AON 40 (Fig. 4A). On the contrary, AONs 55, 66 and 83 were non-inhibitory. These results were confirmed by the reduction of VP1 intensity in a western blot assay using cytoplasmic cell extracts (Fig. 4B). The specific inhibition of AON 40 may be attributed to the pairing of the AONs to the IRES sequence adjacent to *cre* (Fig. 1A), an element necessary for viral replication [14].

**Figure 5:**
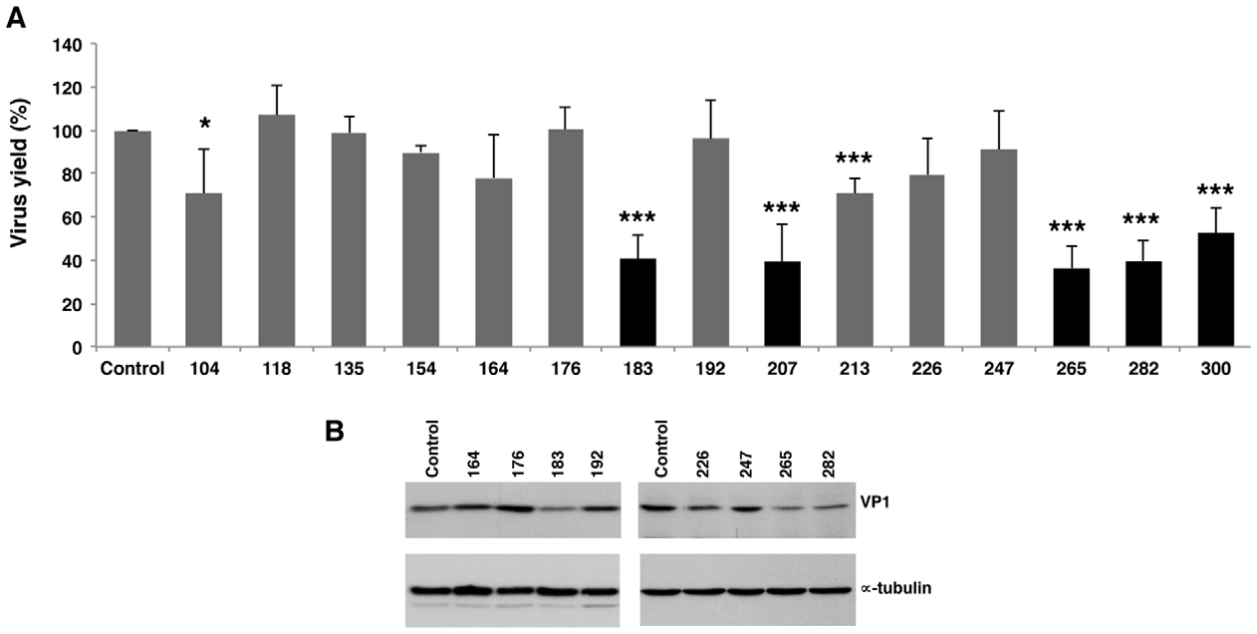
Analysis of virus yield in the presence of 2′OMe AONs complementary to domain 3. (A) FMDV RNA was annealed with the indicated AONs or a scramble (SCR), prior to transfect confluent BHK-21 cell monolayers in duplicate. Virus yield was determined as PFU/ml in the supernatant 24 hpt, and made relative to the value obtained in the control RNA alone. Values represent the mean and standard deviation of triplicate assays (*** *P*<0.01; ** *P*<0.05: * *P*<0.1). (B). Western blot of representative examples of viral protein VP1 accumulated in BHK-21cells 24 hpt; anti-tubulin was used as a loading control.

The inhibitory capacity of AONs complementary to domain 3 is shown in Fig. 5. Gross differences were observed with molecules targeting the basal stem. While AONs 265, 282 and 300 inhibited virus yield (36%, 39% and 52%, respectively), no inhibitory effect was detected with AONs 104, 118, 135, and 154. The reason for these differences is not known. Interestingly, virus yield analysis revealed that AONs 183 and 207, complementary to the GNRA and the RAAA stem-loops respectively (Fig. 3), reduced FMDV RNA infectivity to about 40% (Fig. 5A). However, AONs 164 and 176, complementary to the sequences immediately upstream of the GNRA motif, did not affect virus yield. Moreover, the 3′region of the GNRA and RAAA motifs were resistant to the effect of AONs 192 and 213. Differences in the efficiency of VP1 viral protein synthesis 24 hpt were confirmed by western blot (Fig. 5B). The structural organization of the FMDV IRES that depends on interactions involving the GNRA motif and some nucleotides of the adjacent RAAA stem-loop [16], [26] may form part of the basis of these differences.

**Figure 6:**
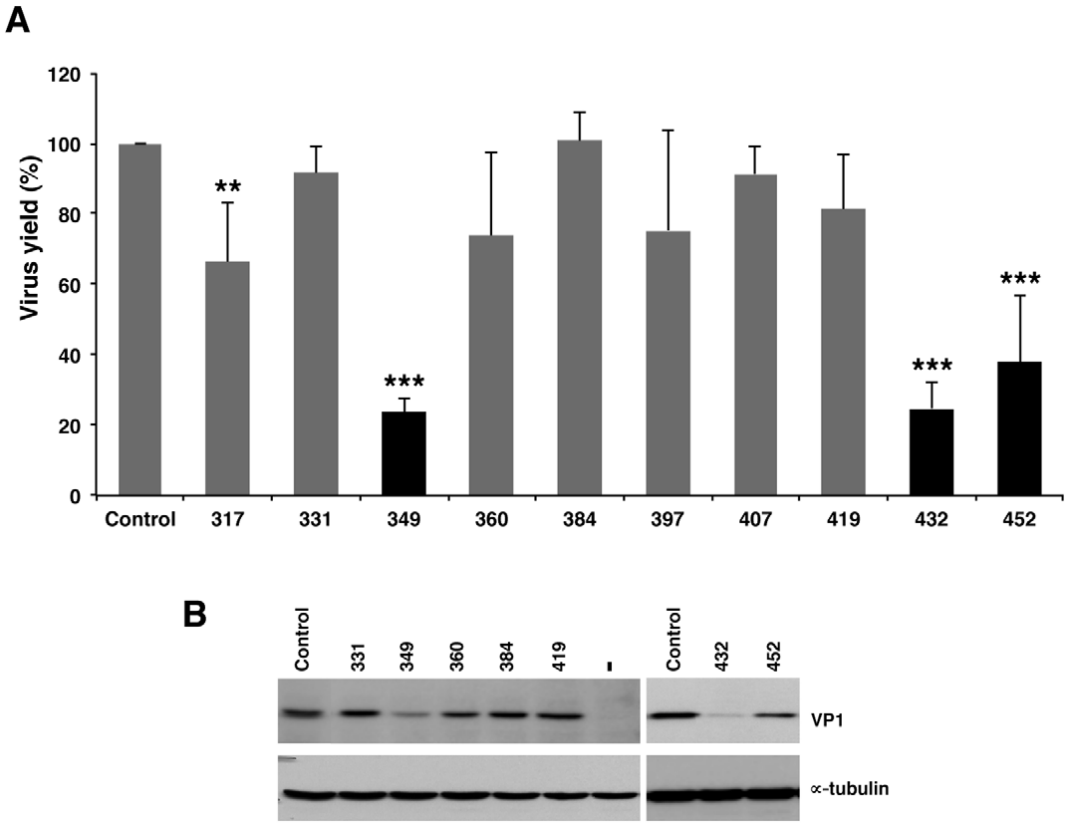
Effect of 2′OMe AONs complementary to domain 5 on virus multiplication. (A) FMDV RNA was annealed with the indicated AONs and a scramble (SCR), prior to transfect confluent BHK-21 cell monolayers in duplicate. Virus yield was determined as PFU/ml in the supernatant 24 hpt, and made relative to the value obtained in the control RNA. Values represent the mean and standard deviation of triplicate assays (*** *P*<0.01; ** *P*<0.05). (B). Western blot of representative examples of viral protein VP1 accumulated in BHK-21cells 24 hpt; anti-tubulin was used as a loading control.

AONs complementary to domain 4 behaved in a different way. With the exception of AON 349 for which a severe decline in virus yield (23% relative to the control RNA) was noticed (Fig. 6A), the yield observed in the presence of AONs 317, 331, 360, 384, 397, 407 and 419 were all above 60%, indicating that viral multiplication was not affected if AONs were pre-annealed to this IRES region. Domain 5, however, did not tolerate the disruption caused by AONs presumably due to the blocking of binding sites of RNA-binding proteins [23], [43]. Specifically, the inhibition was slightly more pronounced in the hairpin (432, 24%) than in the single-stranded region (452, 32%) (Fig. 6A). The inhibition of protein synthesis by AONs complementary to domains 4 and 5 was also confirmed by a decrease in the amount of viral proteins shown in the western blot (Fig. 6B).

**Figure 7:**
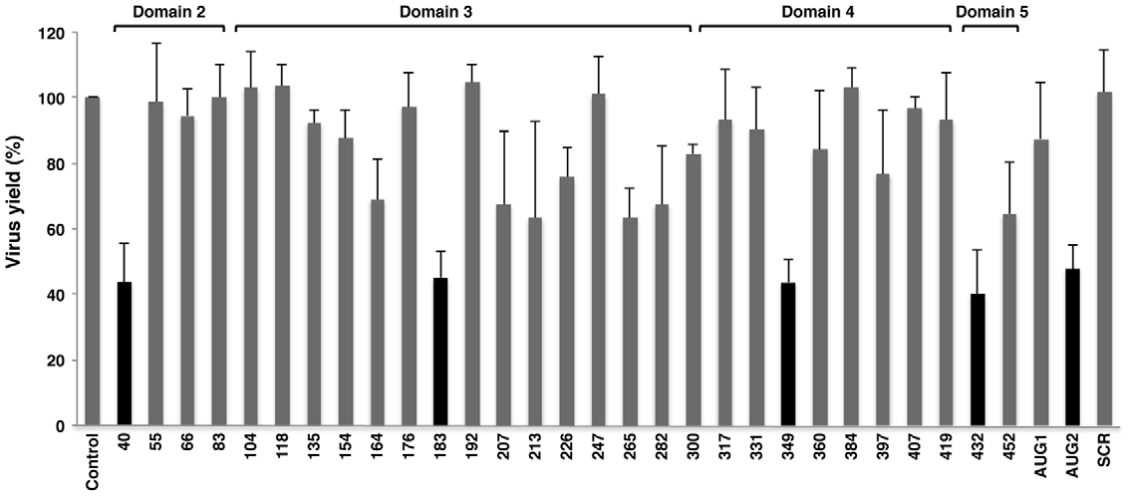
Monitoring time-effect of virus yield induced by 2′OMe AONs complementary to the IRES region. FMDV RNA was annealed with the indicated AONs and a scramble (SCR), prior to transfect confluent BHK-21 cell monolayers in duplicate. Virus yield was determined as PFU/ml in the supernatant 48 hpt, and made relative to the value obtained in the control RNA. Values represent the mean and standard deviation of triplicate assays. A black bar denotes inhibitory capacity (*** *P*<0.01) relative to control transfection.

The capacity of AONs to reduce viral replication was further investigated by extending the incubation of the cells up to 48 hpt. Compared to control RNA, the virus yields revealed that five oligonucleotides (40, 183, 349, 432 and AUG2) remained inhibitory 48 hpt (Fig. 7) (*P*<0.01). It is important to note that these AONs showed the highest potency in inhibiting the viral multiplication 24 hpt, with virus yields below 40%. The inhibition noticed at 48 hpt indicated the potential of the AONs to inhibit viral replication despite the growing number of viral RNA molecules during the viral multiplication process.

### Interference of IRES activity in vitro denotes differences with cultured cells

**Figure 8:**
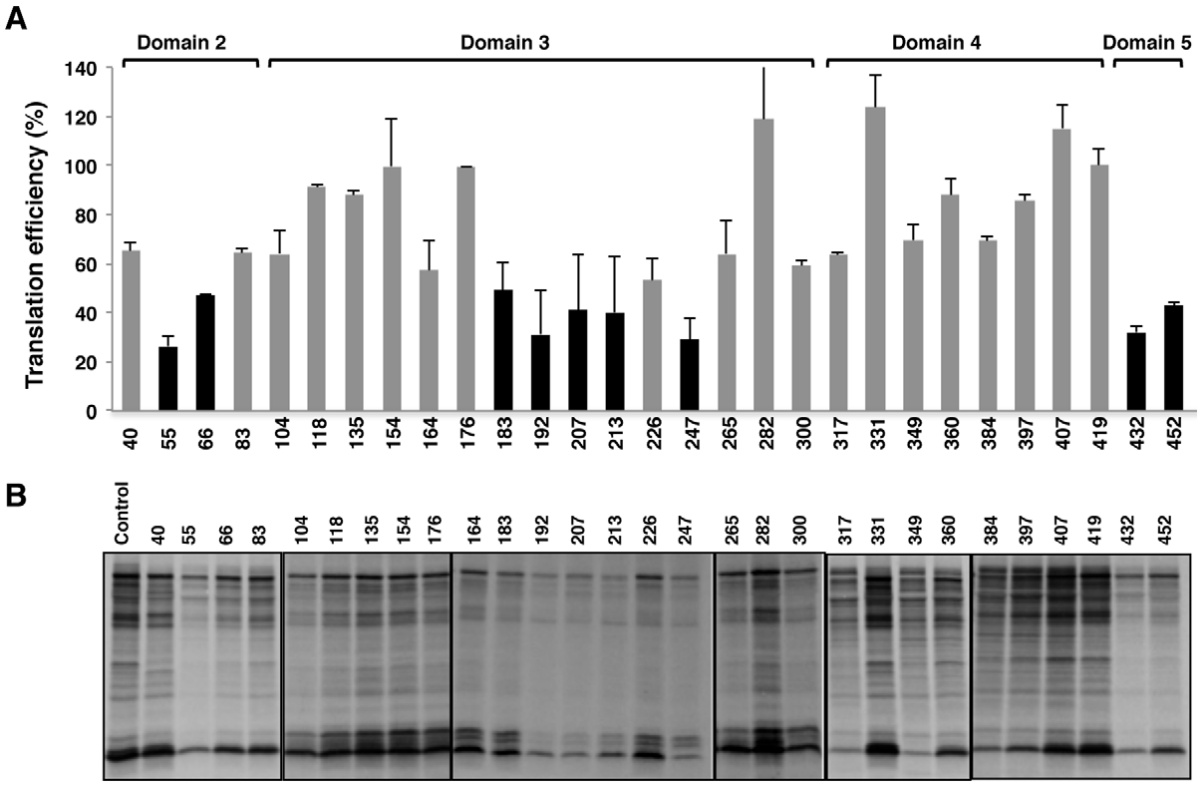
Changes in FMDV RNA translation efficiency *in vitro* induced by 2′OMe AONs. (A). Autoradiograph of translation products obtained from FMDV RNA annealed, or not, with the indicated AONs in reticulocyte lysates. Translation assays were conducted as described in Material and Methods. (B). The intensity of each lane was determined by densitometry and made relative to the intensity of the lane without AON, which was set at 100%. Data correspond to the mean and standard deviation of the mean of three independent assays. A black bar denotes inhibitory capacity relative to control RNA.

After revealing the differential impact of the AUG1 and AUG2 oligonucleotides to down-regulate IRES activity, this work sought to further uncover the competence of AONs to intervene in the role of the IRES within a full-length FMDV genome using an *in vitro* translation system. The inhibition of polyprotein translation *in vitro* induced by AONs annealed to the FMDV RNA varied depending on each molecule (Fig. 8A, B). AONs 55 and 66, complementary to domain 2, induced a decrease in translation efficiency to 26% and 47%, respectively, relative to the control RNA. However, the stem of domain 2 was able to withstand the presence of AONs 40 and 83 (65% and 64%, respectively).

Variations were similarly observed with fifteen AONs complementary to the central IRES domain, with most of the inhibitions noted in molecules complementary to the apical region. In terms of IRES activity, inhibitions were prominent when conserved motifs GNRA, RAAA and C-rich motif were disturbed (ranging from 49 to 29%, with AONs 183, 192, 207, 213 and 247). In contrast, AONs targeting the stem were non-inhibitory (ranging from 64 to 118% with AONs 104 and 282, respectively). The inhibition observed by blocking the apical region of the central domain, reflected the susceptibility of the IRES conserved motifs and revealed the crucial role of the central domain in the translation process.

A striking result was noted in the lack of inhibition exerted by AONs complementary to domain 4, with values above 60% (Fig. 8A). However, and in agreement with the inhibition observed in transfected cells (Fig. 6A), the AONs 432 and 452 were strong inhibitors of protein synthesis.

### Interference of virus multiplication by AONs AUG1 and AUG2

**Figure 9:**
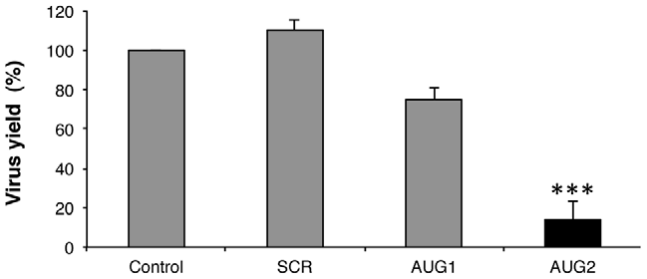
Determination of FMDV virus titer after treatment with SCR, AUG1 and AUG2 AONs. BHK-21 cells were treated with AONs, or mock-treated, 3 h before infection with FMDV C-S8 at 0.1 PFU/ml, in duplicate. After 1 h of adsorption, the AONs were added back to the medium. Virus titer was determined in the supernatant of infected cells 6 hpi in triplicate plaque assays. Values represent the virus yield relative to that observed in the mock-treated cells, which was set at 100%. A black bar denotes inhibitory capacity (*** *P*<0.01) relative to control infection.

To extend our analysis of AON inhibition to virus infection, we used BHK-21 cell monolayers treated with AONs SCR, AUG1, AUG2 and mock-treated to carry out a virus infection as described in Material and Methods. Virus titer determined at time 0 was lower than 10 PFU/ml in all samples. However, the virus titer determined 6 hpi differed strongly between AUG1 and AUG2, while there was no effect of the SCR relative to the control mock-untreated cell monolayer (Fig. 9). The results indicated a significant inhibition in the case of cells treated with AUG2 (<20% relative to the control untreated cells, P<0.01), while the decrease in virus titer in the presence of AUG1 was about 80% of the control cell monolayer. These results are in full agreement with the data obtained in infectious RNA transfections, manifesting a similar inhibitory effect in cells transfected with FMDV RNA than in cells infected with FMDV virus particles.

## Discussion

Here we have made use of 2′OMe AONs complementary to the FMDV IRES-AUG translation start region to explore the capacity of these molecules to interfere viral RNA translation, and thus, viral multiplication. Specifically, we were interested in analyzing the response of the IRES-AUG region in the context of the viral RNA for several reasons. First, to take into account the *cis*-acting elements in the viral RNA that may contribute to IRES function that are absent in reporter sequences and second, to establish a comparison between the responses observed in cell-free systems and in tissue culture cells.

Our results reinforce the main role of AUG2 in viral RNA translation in tissue culture cells. This is supported by the strong inhibition of the virus yield measured 24 hpt with the AON complementary to AUG2, while that complementary to AUG1 only partially reduced virus multiplication (Fig. 1B). In both cases, the inhibition observed was dose-dependent and sequence specific (Fig. S1). These results are consistent with a preferential use of AUG2 in infected cells [39], as well as with the higher inhibitory capacity of antisense oligoribonucleotides targeting AUG2 [36], independently of using FMDV RNA-transfected cells or FMDV infection (Fig. 9). The reasons that would explain the different behavior of AUG1 and AUG2 AONs observed between *in vitro* translation and transfected cells are unknown; however they support a different accessibility of these two regions to external molecules despite being only 84 nt apart. Further, our results support the need to use *in vivo* assays to validate the interference of small molecules in viral gene expression.

The interference observed with a collection of thirty-two customized AONs targeting the entire IRES region revealed large differences in the capacity of these molecules to inhibit viral gene expression, and therefore multiplication of the viral RNA. Specifically, oligonucleotides complementary to domain 2, the apical region of domain 3, the basal region of domain 4 and domain 5 were the most efficient inhibitors in transfected cells, which were still detected 48 hpt (Fig. 7). This inhibition was confirmed when the viral protein VP1 accumulated in the cell cytoplasm prior to cell detachment was measured by immunodetection, indicating that the low virus yield was a consequence of a decrease in viral RNA translation. In full agreement with these results, phosphorothioate derivatives targeting some of these regions were inhibitors of virus multiplication (Table S1).

Annealing of 2′OMe AONs to FMDV RNA prior to program *in vitro* translation indicated a differential response in some, but not all, IRES regions. Specifically, AONs 183, 207, 432 and 452 were inhibitory (as observed in transfected cells). However, several AONs complementary to the apical region of domain 3 were inhibitors *in vitro* but not in living cells. In agreement with this result, differences in DMS and amino-methyl psoralen accessibility to naked FMDV IRES or RNA expressed in the cell cytoplasm were already noticed [24]. Since RNA structure of this particular IRES region is constrained by tertiary interactions [16], [26], it is likely that the composition of the cell-free system differs in cofactors influencing RNA structure. Further, the differences in gene expression observed with the FMDV RNA when transfected in susceptible cells or translated *in vitro* suggested important conformational changes, which may depend on the interaction of the IRES with transacting factors present in different concentrations in the cell cytoplasm. In addition, interactions with other *cis*-acting RNA elements such as the FMDV 3′UTR [44], modification of host factors in infected cells [45]–[50], or the specific intracellular environment of viral replication [51]–[54] may differentially affect expression in these systems. However, since hamster BHK-21 or swine IBRS-2 cell lines are known to have an inactive type I IFN system in response to FMDV infection [55], [56] it is unlikely that the differences observed between the in vitro lysates and the transfected cells are due to type I interferon production.

As previously mentioned, domains 4 and 5 provide the binding site for eIFs and IRES-transacting factors (ITAFs) controlling internal initiation of translation [38], [43], [57]. Disruptions caused by the AONs targeting domain 5 were not tolerated, as reflected by the reduced infectivity of FMDV RNA in the presence of the AON complementary to the hairpin (432) and the single-stranded region (452) (Figs. 6A and 7). The inhibition induced by AONs complementary to domain 5 had similarities to those noted by AONs 40, 183 and 349, complementary to domain 2, the apical region of domain 3, or domain 4 (Fig. 3). Another possibility to explain the inhibition noticed in domain 5 could be the steric block caused by the AONs, interfering in the landing site of the translational machinery.

Our results are in partial disagreement with a previous study that used morpholino oligomers targeting domain 5, AUG1 and AUG2 to inhibit FMDV multiplication [35]. Furthermore, the concentration required to inhibit viral multiplication was higher (1–5 µM) and the interference produced by the morpholino complementary to domain 5 was lower than that of AUG1 or AUG2. Other IRES regions were not analyzed in the work by Vagnozzi et al. [35]. Morpholino-modified RNAs effectively inhibited replication of poliovirus [58], West Nile virus [59], dengue virus [60], or severe acute respiratory syndrome coronavirus [61], among other positive strand RNA viruses. More recently locked nucleic acid-based (LNA) RNAs were shown to be effective inhibitors of HCV [62]. Other RNA-based antiviral strategies were able to suppress virus multiplication in cell culture, as illustrated for HCV [63], [64], FMDV [65] or poliovirus [58], [66].

In summary, we have taken advantage of the stability of 2′OMe AONs to explore the accessibility of the entire IRES-AUG region in the context of the viral RNA. Overall, we have identified critical regions of the IRES element that led to a significant decrease of virus yield when targeted with 2′OMe AONs. Identification of these regions in tissue culture cells by monitoring the reduction in viral protein translation or reduced virus multiplication emphasizes the relevance of certain viral RNA region in controlling viral gene expression. In addition, they open up new avenues to develop novel tools aimed to inhibit viral infection.

## Materials and Methods

### RNA synthesis

The plasmid pMT28, encoding a cDNA copy of FMDV C-S8c1 genome inserted into pGEM-1 under the control of the SP6 promoter was described previously [42]. Following NdeI linearization FMDV RNA was transcribed *in vitro* using SP6 RNA polymerase, as described [8].

### 2′O-methyl antisense oligoribonucleotides design

Thirty-two 2′O-Methyl antisense oligoribonucleotides designed to hybridize with the IRES, AUG1 and AUG2 of FMDV RNA (Table 1), with 2′O-methyl instead of the 2′OH of the ribose in the four flanking nucleotides, were purchased from SIGMA. For simplicity, 2′OMe AONs are named by the IRES nt position which is complementary to the 5′ end of each oligoribonucleotide (see Fig. 3). A scrambled sequence of 16 nt, with 50% GC content, was included as specificity control. The scrambled sequence was checked with NCBI-BLAST software (http://blast.ncbi.nlm.nih.gov/) to prevent any possible match in the FMDV RNA or the host cellular RNAs. MFold (http://www.tbi.univie.ac.at/~ivo/RNA/) and oligo analyzer (http://eu.idtdna.com/analyzer/applications/oligoanalyzer/) were used to predict the secondary structure of each antisense oligoribonucleotide to prevent self-dimerization, formation of stable hairpins, and to optimize its hybridization to the IRES region avoiding perfect hairpin targets deduced from RNA probing data [16]–[18], [25]–[26].

### Annealing of 2′OMe AONs with the RNA and in vitro translation

The optimal inhibitory condition was determined using a concentration range (0.1, 1, 3 and 6 µM) of two AONs, AUG and SCR, annealed to RNA (250 ng) for 20 min at 37°C (data not shown). *In vitro* translation was performed by adding the RNA annealed with AON to a reaction mix containing 6.5 µl of nuclease-treated rabbit reticulocyte lysates (RRL) (Promega), 1 µl (1 mM) amino acid mix less methionine and 0.5 µl (6 µCi) ^35^S-methionine in a final volume of 10 µl. Translation reaction was carried out at 30°C for 60 min. The reaction was treated with 1 µl of RNase A, incubated for 10 min at room temperature and fractionated in polyacrylamide gel with sodium dodecyl sulfate (SDS-PAGE) [11]. Inhibition of viral polypeptides synthesis was calculated by dividing the intensity of total polypeptides translated in the presence of the 2′OMe AON to that of the control RNA without ASO. Subsequent assays were carried out using 6 µM 2′OMe AON, annealed to the target RNA at 37°C, 20 min.

### RNA transfection

The infectivity of viral RNA synthesized *in vitro* was determined using increasing amounts of FMDV RNA (25 pg to 2 ng) to transfect BHK-21 cell monolayers (5×10^5^ cells); virus yield was titrated 24 hpt in fresh cell monolayers. Typically, virus yield from 50 pg of FMDV RNA yielded about 1.6×10^3^ plaque forming units (PFU)/ml. In the RNA infectivity assay, and prior to RNA transfection, 50 pg of *in vitro* synthesized FMDV RNA was annealed with each oligonucleotide (10 nM) for 20 min at 37°C. Triplicates of BHK-21 cells grown in 35 mm well dishes, washed three times with Dulbeccós modified Eagle's medium (DMEM), were transfected with a mixture of lipofectine-RNA annealed with each AON in 0.5 ml of DMEM, as described [9]. Three hours post-transfection (hpt), cell monolayers were washed with DMEM three times, and 2 ml of fresh DMEM supplemented with 5% FCS was added. At 24 and 48 hpt, 200 µl supernatant was collected. Cytoplasmic cell extracts were prepared for the determination of VP1 viral protein from transfected cells 24 hpt prior to cell detachment, using lysis buffer (100 µl of 50 mM Tris-HCl, pH 7.8, 120 mM NaCl, 0.5% NP40) and centrifuged at 14,000 RPM for 5 min to remove cellular debris. Transfection experiments were repeated at least 3 times.

### Western blot analysis

Equal amounts of total protein prepared from transfected BHK-21 transfected cells were resolved in 10% SDS-PAGE, transferred to a PVDF membrane (Biorad) using a semidry electrotransfer device. VP1 protein was detected using the SD6 mouse monoclonal antibody (1∶1000) [67] followed by goat-anti-mouse secondary antibody coupled to horseradish peroxidase (1∶2000) (Thermo Scientific), and enhanced chemiluminescence (GE Healthcare). After stripping using restore western blot stripping buffer (Thermo Scientific) the same membrane was used to detect α-tubulin as a loading control using anti-tubulin antibody (1∶5000) (Sigma).

### PFU inhibition assay

Virus yield from three independent RNA infectivity assays were titrated in fresh susceptible IBRS-2 cells to determine the capacity of each 2′OMe AON to inhibit PFU. Monolayers of IBRS-2 cells were infected with serial dilutions of the supernatant from transfected BHK-21 cells. One hour after adsorption, the viral inoculum was removed and the cells were washed three times, overlaid with DMEM medium with 0.5% agar supplemented with 2% fetal calf serum. Virus titer (PFU/ml) was scored 24 hours post-infection (hpi) by fixing the cells with 2% formaldehyde solution and stained with 0.3% crystal violet in 2% formaldehyde solution. The viral titer from the 24 and 48 hpt supernatant was determined by counting the viral plaques that developed after 24 hpi [33]. The virus yield was calculated as the mean of PFU/ml of three independent assays of FMDV RNA transfected BHK-21 cells with each 2′OMe AONs relative to the PFU/ml of FMDV RNA transfected without oligonucleotides, which was set at 100%. Cell monolayers were used to determine ASO cytotoxicity by cell staining at the end of the treatment.

### Challenge of AON-treated cells with FMDV virus

BHK-21 cell monolayers (1×10^5^ cells) were incubated with SCR, AUG1 or AUG2 ASO (0.4 μM) or DMEM alone 3 h prior to FMDV infection. Then, cell monolayers were infected with C-S8c1 FMDV at a multiplicity of infection (MOI) of 0.1 PFU/cell. After 1 h of adsorption, the inoculum was removed by 3 washes with DMEM-HCl pH 6 to inactivate free virus, twice with DMEM to restore pH and then, incubated with DMEM supplemented with 2% FCS and the appropriate AON (0.4 μM) for 6 h. Supernatants were collected at 0 and 6 hpi for virus titer determination, as described [33].

## Supporting Information

Figure S1 **Determination of the optimum 2′OMe AON concentration required for virus yield inhibition.** *In vitro* synthetized FMDV RNA (50 pg) was annealed with the indicated concentrations of AONs AUG1, AUG2 or the scramble SCR, prior to transfect confluent BHK-21 cell monolayers in duplicate. Virus yield was determined using fresh cells monolayers as the number of plaque forming units (PFU)/ml in the supernatant 24 hpt as described in Material and Methods, made relative to the control RNA which was set at 100%. Values represent the mean and standard deviation of triplicate assays.
(TIF)

Table S1 **Inhibition of FMDV RNA infectivity by phosphorothionate antisense oligonucleotides.**
(DOCX)

## References

[1] Sobrino F, Saiz M, Jimenez-Clavero MA, Nunez JI, Rosas MF (2001). Foot-and-mouth disease virus: a long known virus, but a current threat.. Vet Res.

[2] Grubman MJ, Baxt B (2004). Foot-and-mouth disease.. Clin Microbiol Rev.

[3] Martinez-Salas E, Ryan M, Ehrenfeld E, Domingo E, Roos R (2010). Translation and protein processing..

[4] King AM, Sangar DV, Harris TJ, Brown F (1980). Heterogeneity of the genome-linked protein of foot-and-mouth disease virus.. J Virol.

[5] Belsham GJ, Brangwyn JK (1990). A region of the 5′ noncoding region of foot-and-mouth disease virus RNA directs efficient internal initiation of protein synthesis within cells: involvement with the role of L protease in translational control.. J Virol.

[6] Kuhn R, Luz N, Beck E (1990). Functional analysis of the internal translation initiation site of foot-and-mouth disease virus.. J Virol.

[7] Martinez-Salas E, Saiz JC, Davila M, Belsham GJ, Domingo E (1993). A single nucleotide substitution in the internal ribosome entry site of foot-and-mouth disease virus leads to enhanced cap-independent translation in vivo.. J Virol.

[8] Lopez de Quinto S, Saiz M, de la Morena D, Sobrino F, Martinez-Salas E (2002). IRES-driven translation is stimulated separately by the FMDV 3′-NCR and poly(A) sequences.. Nucleic Acids Res.

[9] Saiz M, Gomez S, Martinez-Salas E, Sobrino F (2001). Deletion or substitution of the aphthovirus 3′ NCR abrogates infectivity and virus replication.. J Gen Virol.

[10] Forss S, Strebel K, Beck E, Schaller H (1984). Nucleotide sequence and genome organization of foot-and-mouth disease virus.. Nucleic Acids Res.

[11] Lopez de Quinto S, Martinez-Salas E (1999). Involvement of the aphthovirus RNA region located between the two functional AUGs in start codon selection.. Virology.

[12] Andreev DE, Fernandez-Miragall O, Ramajo J, Dmitriev SE, Terenin IM (2007). Differential factor requirement to assemble translation initiation complexes at the alternative start codons of foot-and-mouth disease virus RNA.. RNA.

[13] Martinez-Salas E, Pacheco A, Serrano P, Fernandez N (2008). New insights into internal ribosome entry site elements relevant for viral gene expression.. J Gen Virol.

[14] Mason PW, Bezborodova SV, Henry TM (2002). Identification and characterization of a cis-acting replication element (cre) adjacent to the internal ribosome entry site of foot-and-mouth disease virus.. J Virol.

[15] Luz N, Beck E (1991). Interaction of a cellular 57-kilodalton protein with the internal translation initiation site of foot-and-mouth disease virus.. J Virol.

[16] Fernandez-Miragall O, Martinez-Salas E (2003). Structural organization of a viral IRES depends on the integrity of the GNRA motif.. RNA.

[17] Fernandez-Miragall O, Ramos R, Ramajo J, Martinez-Salas E (2006). Evidence of reciprocal tertiary interactions between conserved motifs involved in organizing RNA structure essential for internal initiation of translation.. RNA.

[18] Martinez-Salas E, Regalado MP, Domingo E (1996). Identification of an essential region for internal initiation of translation in the aphthovirus internal ribosome entry site and implications for viral evolution.. J Virol.

[19] Serrano P, Ramajo J, Martinez-Salas E (2009). Rescue of internal initiation of translation by RNA complementation provides evidence for a distribution of functions between individual IRES domains.. Virology.

[20] Yu Y, Abaeva IS, Marintchev A, Pestova TV, Hellen CU (2011). Common conformational changes induced in type 2 picornavirus IRESs by cognate trans-acting factors. Nucleic Acids Res..

[21] Lopez de Quinto S, Martinez-Salas E (2000). Interaction of the eIF4G initiation factor with the aphthovirus IRES is essential for internal translation initiation in vivo.. RNA.

[22] Pilipenko EV, Pestova TV, Kolupaeva VG, Khitrina EV, Poperechnaya AN (2000). A cell cycle-dependent protein serves as a template-specific translation initiation factor.. Genes Dev.

[23] Martinez-Salas E (2008). The impact of RNA structure on picornavirus IRES activity.. Trends Microbiol.

[24] Fernandez-Miragall O, Martinez-Salas E (2007). In vivo footprint of a picornavirus internal ribosome entry site reveals differences in accessibility to specific RNA structural elements.. J Gen Virol.

[25] Fernandez N, Garcia-Sacristan A, Ramajo J, Briones C, Martinez-Salas E (2011). Structural analysis provides insights into the modular organization of picornavirus IRES.. Virology.

[26] Fernandez N, Fernandez-Miragall O, Ramajo J, Garcia-Sacristan A, Bellora N (2011). Structural basis for the biological relevance of the invariant apical stem in IRES-mediated translation. Nucleic Acids Res..

[27] Hoke GD, Draper K, Freier SM, Gonzalez C, Driver VB (1991). Effects of phosphorothioate capping on antisense oligonucleotide stability, hybridization and antiviral efficacy versus herpes simplex virus infection.. Nucleic Acids Res.

[28] Inoue H, Hayase Y, Iwai S, Ohtsuka E (1987). Sequence-dependent hydrolysis of RNA using modified oligonucleotide splints and RNase H. FEBS letters.

[29] Majlessi M, Nelson NC, Becker MM (1998). Advantages of 2′-O-methyl oligoribonucleotide probes for detecting RNA targets.. Nucleic Acids Res.

[30] Haraguchi T, Nakano H, Tagawa T, Ohki T, Ueno Y (2012). A potent 2′-O-methylated RNA-based microRNA inhibitor with unique secondary structures. Nucleic Acids Res..

[31] Hutvagner G, Simard MJ, Mello CC, Zamore PD (2004). Sequence-specific inhibition of small RNA function.. PLoS Biology.

[32] Gutierrez A, Martinez-Salas E, Pintado B, Sobrino F (1994). Specific inhibition of aphthovirus infection by RNAs transcribed from both the 5′ and the 3′ noncoding regions.. J Virol.

[33] Rosas MF, Martinez-Salas E, Sobrino F (2003). Stable expression of antisense RNAs targeted to the 5′ non-coding region confers heterotypic inhibition to foot-and-mouth disease virus infection.. J Gen Virol.

[34] Bigeriego P, Rosas MF, Zamora E, Martinez-Salas E, Sobrino F (1999). Heterotypic inhibition of foot-and-mouth disease virus infection by combinations of RNA transcripts corresponding to the 5′ and 3′ regions.. Antiviral Res.

[35] Vagnozzi A, Stein DA, Iversen PL, Rieder E (2007). Inhibition of foot-and-mouth disease virus infections in cell cultures with antisense morpholino oligomers.. J Virol.

[36] Gutierrez A, Rodriguez A, Pintado B, Sobrino F (1993). Transient inhibition of foot-and-mouth disease virus infection of BHK-21 cells by antisense oligonucleotides directed against the second functional initiator AUG.. Antiviral Res.

[37] Lopez de Quinto S, Martinez-Salas E (1997). Conserved structural motifs located in distal loops of aphthovirus internal ribosome entry site domain 3 are required for internal initiation of translation.. J Virol.

[38] Lopez de Quinto S, Lafuente E, Martinez-Salas E (2001). IRES interaction with translation initiation factors: functional characterization of novel RNA contacts with eIF3, eIF4B, and eIF4GII.. RNA.

[39] Cao X, Bergmann IE, Fullkrug R, Beck E (1995). Functional analysis of the two alternative translation initiation sites of foot-and-mouth disease virus.. J Virol.

[40] Belsham GJ (1992). Dual initiation sites of protein synthesis on foot-and-mouth disease virus RNA are selected following internal entry and scanning of ribosomes in vivo.. EMBO J.

[41] Lopez de Quinto S, Martinez-Salas E (1998). Parameters influencing translational efficiency in aphthovirus IRES-based bicistronic expression vectors.. Gene.

[42] Garcia-Arriaza J, Manrubia SC, Toja M, Domingo E, Escarmis C (2004). Evolutionary transition toward defective RNAs that are infectious by complementation.. J Virol.

[43] Pacheco A, Reigadas S, Martinez-Salas E (2008). Riboproteomic analysis of polypeptides interacting with the internal ribosome-entry site element of foot-and-mouth disease viral RNA.. Proteomics.

[44] Serrano P, Pulido MR, Saiz M, Martinez-Salas E (2006). The 3′ end of the foot-and-mouth disease virus genome establishes two distinct long-range RNA-RNA interactions with the 5′ end region.. J Gen Virol.

[45] Rodriguez Pulido M, Serrano P, Saiz M, Martinez-Salas E (2007). Foot-and-mouth disease virus infection induces proteolytic cleavage of PTB, eIF3a, b, and PABP RNA-binding proteins. Virology..

[46] Belsham GJ, McInerney GM, Ross-Smith N (2000). Foot-and-mouth disease virus 3C protease induces cleavage of translation initiation factors eIF4A and eIF4G within infected cells.. J Virol.

[47] Gradi A, Foeger N, Strong R, Svitkin YV, Sonenberg N (2004). Cleavage of eukaryotic translation initiation factor 4GII within foot-and-mouth disease virus-infected cells: identification of the L-protease cleavage site in vitro.. J Virol.

[48] Armer H, Moffat K, Wileman T, Belsham GJ, Jackson T (2008). Foot-and-mouth disease virus, but not bovine enterovirus, targets the host cell cytoskeleton via the nonstructural protein 3Cpro. J Virol..

[49] Pineiro D, Ramajo J, Bradrick SS, Martinez-Salas E (2012). Gemin5 proteolysis reveals a novel motif to identify L protease targets. Nucleic Acids Res..

[50] Lawrence P, Schafer EA, Rieder E (2012). The nuclear protein Sam68 is cleaved by the FMDV 3C protease redistributing Sam68 to the cytoplasm during FMDV infection of host cells.. Virology.

[51] Piccone ME, Feng Y, Chang AC, Mosseri R, Lu Q (2009). Identification of cellular genes affecting the infectivity of foot-and-mouth disease virus.. J Virol.

[52] Monaghan P, Cook H, Jackson T, Ryan M, Wileman T (2004). The ultrastructure of the developing replication site in foot-and-mouth disease virus-infected BHK-38 cells.. J Gen Virol.

[53] Knox C, Moffat K, Ali S, Ryan M, Wileman T (2005). Foot-and-mouth disease virus replication sites form next to the nucleus and close to the Golgi apparatus, but exclude marker proteins associated with host membrane compartments.. J Gen Virol.

[54] O'Donnell V, Pacheco JM, LaRocco M, Burrage T, Jackson W (2011). Foot-and-mouth disease virus utilizes an autophagic pathway during viral replication.. Virology.

[55] de Los Santos T, de Avila Botton S, Weiblen R, Grubman MJ (2006). The leader proteinase of foot-and-mouth disease virus inhibits the induction of beta interferon mRNA and blocks the host innate immune response.. J Virol.

[56] Rodriguez-Pulido M, Borrego B, Sobrino F, Saiz M (2011). RNA structural domains in noncoding regions of the foot-and-mouth disease virus genome trigger innate immunity in porcine cells and mice.. J Virol.

[57] Pacheco A, Lopez de Quinto S, Ramajo J, Fernandez N, Martinez-Salas E (2009). A novel role for Gemin5 in mRNA translation.. Nucleic Acids Res.

[58] Stone JK, Rijnbrand R, Stein DA, Ma Y, Yang Y (2008). A morpholino oligomer targeting highly conserved internal ribosome entry site sequence is able to inhibit multiple species of picornavirus.. Antimicrob Agents Chemother.

[59] Deas TS, Binduga-Gajewska I, Tilgner M, Ren P, Stein DA (2005). Inhibition of flavivirus infections by antisense oligomers specifically suppressing viral translation and RNA replication.. J Virol.

[60] Kinney RM, Huang CY, Rose BC, Kroeker AD, Dreher TW (2005). Inhibition of dengue virus serotypes 1 to 4 in vero cell cultures with morpholino oligomers.. J Virol.

[61] Neuman BW, Stein DA, Kroeker AD, Churchill MJ, Kim AM (2005). Inhibition, escape, and attenuated growth of severe acute respiratory syndrome coronavirus treated with antisense morpholino oligomers.. J Virol.

[62] Laxton C, Brady K, Moschos S, Turnpenny P, Rawal J (2011). Selection, optimization, and pharmacokinetic properties of a novel, potent antiviral locked nucleic acid-based antisense oligomer targeting hepatitis C virus internal ribosome entry site.. Antimicrob Agents Chemother.

[63] Wakita T, Moradpour D, Tokushihge K, Wands JR (1999). Antiviral effects of antisense RNA on hepatitis C virus RNA translation and expression.. J Med Virol.

[64] Gonzalez-Carmona MA, Vogt A, Heinicke T, Quasdorff M, Hoffmann P (2011). Inhibition of hepatitis C virus gene expression by adenoviral vectors encoding antisense RNA in vitro and in vivo.. J Hepatol.

[65] de los Santos T, Wu Q, de Avila Botton S, Grubman MJ (2005). Short hairpin RNA targeted to the highly conserved 2B nonstructural protein coding region inhibits replication of multiple serotypes of foot-and-mouth disease virus.. Virology.

[66] Gitlin L, Stone JK, Andino R (2005). Poliovirus escape from RNA interference: short interfering RNA-target recognition and implications for therapeutic approaches.. J Virol.

[67] Rosas MF, Vieira YA, Postigo R, Martin-Acebes MA, Armas-Portela R (2008). Susceptibility to viral infection is enhanced by stable expression of 3A or 3AB proteins from foot-and-mouth disease virus.. Virology.

