# Supplementary figures and images for: Exploring IRES Region Accessibility by Interference of Foot-and-Mouth Disease Virus Infectivity
Source: PLoS One. 2012 Jul 18;7(7):e41382. doi: 10.1371/journal.pone.0041382 (PMC3399821; doi:10.1371/journal.pone.0041382)

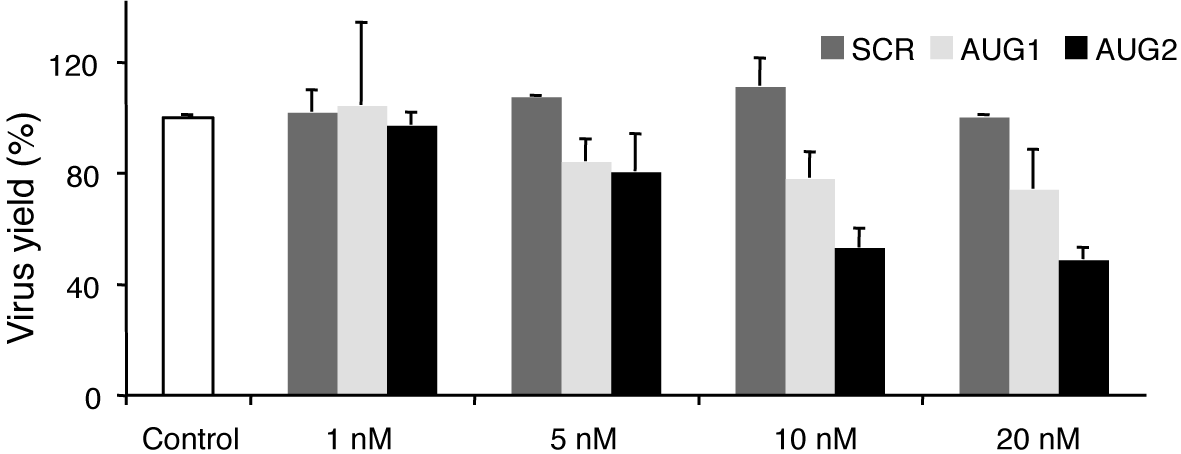

Supplement: Figure S1 — Determination of the optimum 2′OMe AON concentration required for virus yield inhibition. In vitro synthetized FMDV RNA (50 pg) was annealed with the indicated concentrations of AONs AUG1, AUG2 or the scramble SCR, prior to transfect confluent BHK-21 cell monolayers in duplicate. Virus yield was determined using fresh cells monolayers as the number of plaque forming units (PFU)/ml in the supernatant 24 hpt as described in Material and Methods, made relative to the control RNA which was set at 100%. Values represent the mean and standard deviation of triplicate assays. (TIF) [file pone.0041382.s001.tif]
