# Supplementary material for: Exploring IRES Region Accessibility by Interference of Foot-and-Mouth Disease Virus Infectivity
Source: PLoS One. 2012 Jul 18;7(7):e41382. doi: 10.1371/journal.pone.0041382 (PMC3399821; doi:10.1371/journal.pone.0041382)
Supplement: Table S1 — Inhibition of FMDV RNA infectivity by phosphorothionate antisense oligonucleotides. (DOCX) [file pone.0041382.s002.docx]

**Table S1. Inhibition of FMDV RNA infectivity by phosphorothionate antisense oligonucleotides**

| S-ODN | Size | **Sequence (5´- 3´)** | Position | **Virus yield (%)**^a^ |
| --- | --- | --- | --- | --- |
| 193 | 18 | CGTGGGTCCTTGTTACCA | 176-193 | 30 ± 2.4 |
| 190 | 18 | GGGTCCTTGTTACCAAGG | 173-190 | 35 ± 2.5 |
| 203 | 15 | CTTTTGGCCCCGTGG | 189-203 | 52 ± 2.1 |
| 209 | 15 | GCGTGGCTTTTGGCC | 195-209 | 55 ± 4.2 |
| 306 | 19 | GTCACCAGTGTGTGGGTAC | 288-306 | 30 ± 3.3 |
| 101 | 18 | GCGTGGAGCCAAACACAG | 84-101 | 72 ± 6.8 |
| AUG | 25 | GCGTGAATTCCATTTTTCCTGCAGT | 535-559 | 44 ± 3.9 |
| SCR-2 | 25 | TTTCGTTTATAGCTCGAGTTGCCCA |  | 100 |

a: Virus yield was determined in BHK-2 transfected cells using FMDV C-S8 purified viral RNA performed as described [[34](#_ENREF_34)].
